# Evaluation of the effect of magnesium sulphate vs. clonidine as adjunct to epidural bupivacaine

**DOI:** 10.4103/0019-5049.68373

**Published:** 2010

**Authors:** Tanmoy Ghatak, Girish Chandra, Anita Malik, Dinesh Singh, Vinod Kumar Bhatia

**Affiliations:** Department of Anaesthesia, CSMMU (erstwhile KGMC), Lucknow, Uttar Pradesh, India

**Keywords:** Bupivacaine, clonidine, epidural anaesthesia, magnesium sulphate

## Abstract

For treatment of intra and postoperative pain, no drug has yet been identified that specifically inhibits nociception without associated side effects. Magnesium has antinociceptive effects in animal and human models of pain. The current prospective randomised double-blind study was undertaken to establish the effect of addition of magnesium or clonidine, as adjuvant, to epidural bupivacaine in lower abdominal and lower limb surgeries. A total of 90 American Society of Anesthesiology (ASA) grade I and II patients undergoing lower abdominal and lower limb surgeries were enrolled to receive either magnesium sulphate (Group B) or clonidine (Group C) along with epidural bupivacaine for surgical anaesthesia. All patients received 19 ml of epidural bupivacaine 0.5% along with 50 mg magnesium in group B, 150 mcg clonidine in Group C, whereas in control group (Group A), patients received same volume of normal saline. Onset time, heart rate, blood pressure, duration of analgesia, pain assessment by visual analogue score (VAS) and adverse effects were recorded. Onset of anaesthesia was rapid in magnesium group (Group B). In group C there was prolongation of duration of anaesthesia and sedation with lower VAS score, but the incidence of shivering was higher. The groups were similar with respect to haemodynamic variables, nausea and vomiting. The current study establishes magnesium sulphate as a predictable and safe adjunct to epidural bupivacaine for rapid onset of anaesthesia and clonidine for prolonged duration of anaesthesia with sedation.

## INTRODUCTION

Epidural anaesthesia is a safe and inexpensive technique with the advantage of providing surgical anaesthesia and prolonged postoperative pain relief. It is also an effective treatment of operative pain blunts autonomic, somatic and endocrine responses. It has become a common practice to use polypharmacy approach for treatment of intra and postoperative pain, because no drug has yet been identified that specifically inhibits nociception without associated side effects.[1] Research continues concerning different techniques and drugs that could provide better surgical anaesthesia and postoperative pain relief.

Magnesium is the fourth most plentiful cation in our body. It has antinociceptive effects in animal and human models of pain.[2] It has been mentioned in a systematic review that it may be worthwhile to further study the role of supplemental magnesium in providing perioperative analgesia, because this is a relatively harmless molecule, is not expensive and also because the biological basis for its potential antinociceptive effect is promising.[3] These effects are primarily based on physiological calcium antagonism, that is voltage-dependent regulation of calcium influx into the cell, and noncompetitive antagonism of N-methyl-D-aspartate (NMDA) receptors.[1]

Clonidine is centrally acting partial α2 adrenergic agonist with selectivity ratio 200 : 1.[4] It inhibits voltage gated Na^+^ channels and suppresses the generation of action potentials in tonic firing dorsal horn neurons, causing analgesia.[5] It produces antinociception by stimulating postsynaptic α_2_ adrenergic receptors in the dorsal horn of spinal cord. This mimics the effects of nor-adrenaline which is released from the descending inhibitory pathways in the central nervous system. Thus, decreased activity of second-order neurons and wide dynamic range neurons in the dorsal horn occurs,[5,6] which in turn attenuates the input from peripheral nociceptive Aδ and C fibers.

No clinical studies have examined the effect of magnesium sulphate administered epidurally as an adjunct to epidural bupivacaine. We, therefore, conducted a prospective, randomised, double blind, controlled clinical study to compare the effects of magnesium sulphate vs. clonidine co administered epidurally as adjunct to bupivacaine.

## METHODS

After obtaining institutional ethical committee approval and written informed consent, 90 patients undergoing elective lower abdominal and lower limb surgeries aged 18 to 60 years of either gender, belonging to American Society of Anesthesiology physical status I and II, with ±20% of ideal body weight, were recruited from a group of 121 potential candidates. Patients for whom central neuraxial block is contraindicated, those with history of adverse reaction to any study medication, history of analgesic use, chronic pain syndrome and where communication difficulties preventing reliable assessment were excluded from this study.

Randomisation was done by using a computer-derived random-number sequence and sealed opaque envelopes, and all investigators were kept unaware of the envelope details throughout the whole study period.

After intravenous access, an infusion of ringer’s lactate (20 ml/kg) comprised preloading. All patients had an epidural anaesthetic with an 18 G Tuohy needle. The epidural space was identified at L_2-3_ or L_3-4_ using a loss of resistance technique under strict asepsis, and a 20 G epidural catheter was then advanced for 3 to 5 cm into the epidural space. Correct placement of epidural catheter was verified with a test dose of 3 ml epidural lignocaine 2% with adrenaline (1 : 2,00,000). The patients were then divided randomly into following three groups according to the epidural medications they received:

Group A (control group): bupivacaine 0.5% (19 ml) + saline 0.9% (1 ml).

Group B: bupivacaine 0.5% (19 ml) + magnesium sulphate 50 mg (in 1 ml 0.9% saline)

Group C: bupivacaine 0.5% (19 ml) + clonidine 150 mcg (1 ml).

Sensory block was assessed bilaterally by using analgesia to pin prick with a short hypodermic needle in midclavicular line. Motor blockade was assessed by using modified Bromage scale (0: no motor block; 1: inability to raise extended legs; 2: inability to flex knees; 3: inability to flex ankle joints).[7]

Monitoring consisted of heart rate, noninvasive arterial blood pressure and SpO_2_ measurements in three groups preoperatively, intraoperatively and during shifting. Hypotension was defined as systolic blood pressure <90 mmHg or >30% decrease in baseline values. Tachycardia was defined as heart rate >100/min and bradycardia was defined as heart rate <60/min.

Sedation was assessed on a four point scale[8] (Grade 0, awake and alert; 1, mildly sedated, easily aroused; 2, moderately sedated, aroused by shaking; 3, deeply sedated, difficult to be aroused by physical stimulation).

The patients were asked to evaluate their pain on standard 100 point visual analogue pain scale (VAS 0 = no pain, VAS 100 = worst possible pain). In the event of pain, (VAS ≥40), both intraoperatively as well as postoperatively, a bolus of epidural bupivacaine 0.25% (8 ml) was administered by the anaesthesiologist inside the operation theatre and the nursing staff in the recovery room.

Time to two segment regression to first epidural top up requirement and occurrence of adverse effects, if any, were recorded.

All statistical analyses were performed using SPSS for windows 15.0. Continuous variables were tested for normal distribution by the Kolmogorov-Smirnov test. Parametric data were compared using analysis of variance (ANOVA) within group comparisons at different time intervals assessed by using paired t-test. All the categorical data were compared by using chi-square test. A sample size of 20 patients per group was needed to detect an intergroup difference of at least 20% (α= 0.01, two-sided, power = 90%) with two sample t-test.[9] A value of *P*<0.05 was considered statistically significant. The results are expressed as mean (SD).

## RESULTS

**Figure 1:**
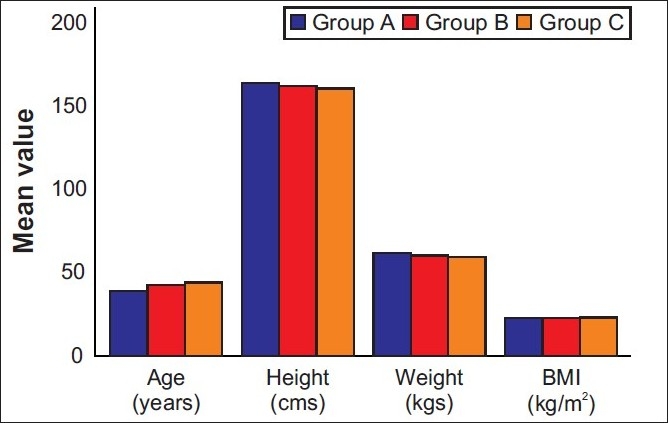
Patient characteristics in the three groups. Data are given as mean (SD)

**Figure 2:**
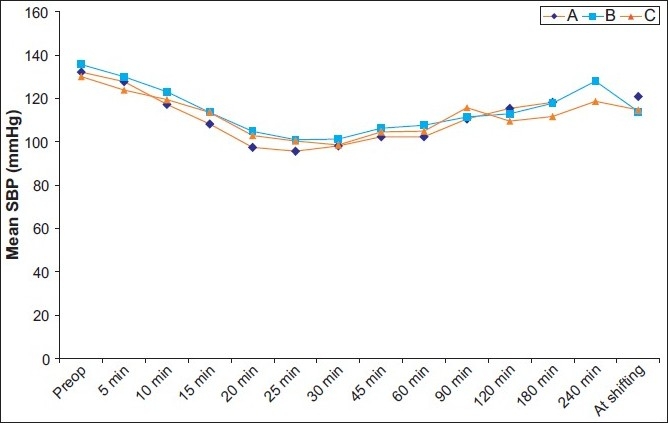
Systolic arterial blood pressures (SAP) in the operative period. There were no significant differences between groups. Data are given as mean (SD)

There were no differences in age, height, body weight, body mass index (BMI) between the groups [Figure 1]. These groups were similar in the maximal dermatome height achieved. No difference in the quality of sensory and motor block before and during the surgery was noted between groups. Systolic, diastolic arterial blood pressures, heart rates and oxygen saturations remained stable, and there was no significant difference between the groups. Systolic arterial blood pressures were similar in this period [Figure 2].

Time to achieve T6 block was least in epidural magnesium adjuvant group (11.80 ± 3.21 minutes) and highest (18.73 ± 2.79 minutes) in control group, whereas it was 16.93 ± 3.43minutes in clonidine group of patients. The difference between the groups was statistically significant. (F = 19.496; *P*<0.05).

**Figure 3:**
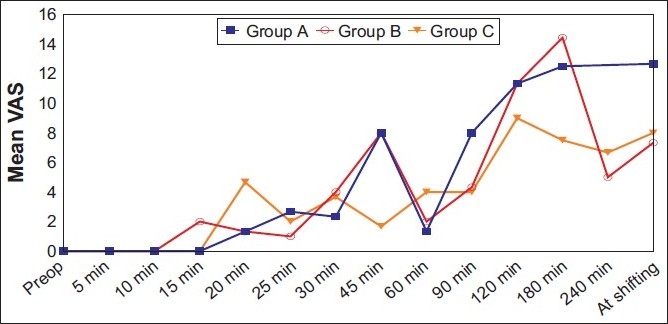
Intergroup comparison of intensity of operative pain as measured using a VAS at different time intervals. The difference in intensity of operative pain between groups was statistically significant at 45 minutes (*P* < 0.05) by using two-way repeated-measures analysis of variance (ANOVA). Data are given as mean (SD)

Wide variation in pain scores were seen throughout the study period. However, statistically significant intergroup differences were seen only at 45 minutes. At this time, clonidine group showing significantly lower pain scores as compared with control group and magnesium group [Figure 3].

The time from epidural medication to first epidural top up was maximum (180.33 ± 29.97 minutes) in clonidine group followed by magnesium group (161.67 ± 30.10 minutes) and with a minimum (150.67 ± 35.80 minutes) in control group of patients. The differences among groups were significant (*P*<0.05).

The time from epidural medication to two segment regression ranged from 123.00 ± 28.08 minutes in control Group (Group A) to 145.33 ± 27.74 minutes in clonidine Group (Group C), with an intermediate value (130.33 ± 33.94 minutes) in magnesium Group (Group B) patients. The differences in time from epidural medication to two segment regression among groups were not statistically significant (*P*>0.05) [Table 1].

**Table 1:** Time to achieve neuraxial block landmarks

Landmark	Group A N=30		Group B N=30		Group C N=30		Statistical significance^*^	
	Mean	SD	Mean	SD	Mean	SD	“F”	“*P*”
Time taken to achieve T6 block (min)	18.73	2.79	11.80	3.21	16.93	3.43	19.496	<0.001^*^
Time to 1^st^ epidural top-up (min)	150.67	35.80	161.67	30.10	180.33	29.97	3.280	0.047
Time to 2^nd^ segment regression (min)	123.00	28.08	130.33	33.94	145.33	27.74	2.152	0.129

Shivering occurred in four (13.3%) control group and seven (33.3%) clonidine group patients, whereas no patients in magnesium group (Group B) suffered from shivering during this study. The differences among groups were statistically significant (*P*<0.05).

Sedation was observed in seven patients (23.3%) in clonidine group (Group C), which is statistically significant (*P*<0.05) [Table 2].

**Table 2:** Adverse effects in different groups

Characteristics	Group A (n=30)		Group B (n=30)		Group C (n=30)		Statistical significance^*^	
	No.	%	No.	%	No.	%	*χ*^2^	*P*
Hypotension	24	80	19	63.33	22	73.33	2.105	0.349
Bradycardia	6	20	4	13.33	10	33.3	3.60	0.165
Nausea and vomiting	2	6.7	2	6.7	6	20.0	3.60	0.165
Shivering	4	13.3	0	0	7	33.3	7.664	0.022
Sedation	0	0	0	0	7	23.33	15.181	<0.001

## DISCUSSION

The results of this study show that the addition of magnesium, a competitive NMDA antagonist, as adjuvant to epidural bupivacaine reduces time of onset and establishment of epidural block upto T_6_ level. With addition of clonidine, centrally acting α2 agonist prolongs anaethesia duration along with significant sedation.

No study using epidural magnesium sulphate as an adjunct to epidural bupivacaine has been done till today. Zand *et al*.[10] showed that the time to onset of sensory block in L1 was 17.12 ± 2.18 minutes in group of patients receiving a total of 18 ml plain 0.5% bupivacaine, and their study time to onset of sensory block in T10 was 24.9 ± 2.54 minutes. Our study also shows onset to T_6_ level was 18.73 ± 2.79 minutes in control group. Early onset may be due to use of a test dose of 3 ml epidural lignocaine 2% with adrenaline (1 : 2,00,000).

Noxious stimulation leads to the release of neurotransmitters, which bind to various subclasses of excitatory amino acid receptors, including NMDA receptors. Activation of these receptors leads to calcium entry into the cell and initiates a series of central sensitization such as wind-up and long-term potentiation in the spinal cord in the response of cells to prolonged stimuli.[11] NMDA receptor signaling may be important in determining the duration of acute pain.[12] Magnesium blocks calcium influx and noncompetitively antagonizes NMDA receptor channels.[13] Noncompetitive NMDA receptor antagonists can have an effect on pain when used alone.[14] Co administration of epidural magnesium for postoperative epidural analgesia provided a pronounced reduction in patient-controlled epidural fentanyl consumption without any side effects.[14]

Clonidine induces dose-dependent spinal cord antinociception, mainly through stimulation of α_2_-adrenoceptors in the dorsal horn, mimicking the activation of descending inhibitory pathways.[15]

Most of these studies showed that systemic administration of magnesium is associated with smaller analgesic requirement and less discomfort in the postoperative period.[16,17] In recent years, intrathecal administration of magnesium has been reported as an effective analgesic and as an adjunct to intrathecal lignocaine anaesthesia.[18] It is possible that analgesic effect of magnesium occurred at the supra-spinal level and might be related to its systemic absorption. But Ko *et al*.[19] failed to observe postoperative analgesic effect with 50 mg/kg intravenously administered magnesium sulphate, and they reported that perioperative administration of magnesium did not increase cerebro spinal fluid (CSF) magnesium concentration. When compared with these doses, our epidural dose is too low for the systemic effect. Although there is no study about the physicochemical properties of magnesium in relation to its penetration to spinal meninges, another probable mechanism for epidural usage may be related to the diffusion of magnesium from the dura. The addition of intrathecal magnesium 50 mg to spinal anaesthesia prolongs the period of anaesthesia without additional side-effects.[20]

Motor and sensory blockade effects of local anaesthetics are enhanced by clonidine. The effects of clonidine on the prolongation of nerve blockade are clearly dose-dependent.[21] We also found in our study that the time gap between initial epidural medication and the time to first epidural top up was highest (180.33 ± 29.97 minutes) in clonidine group followed by magnesium group (161.67 ± 30.10 minutes), and it was 150.67 ± 35.80 minutes in control group of patients. The differences among groups were statistically significant (*P*<0.05).

After neuraxial or systemic administration, clonidine affects arterial blood pressure in a complex manner because of opposing actions at multiple sites. The α_2_-adrenergic agonists reduce sympathetic drive and arterial blood pressure through effects at specific brainstem nuclei and sympathetic preganglionic neurons in the spinal cord. Eisenach *et al*.[22] showed that 160 *µ*g clonidine decreases arterial blood pressure by 18% and reduces heart rate by 5to 20%, and concluded that epidural clonidine does not induce haemodynamic instability. In our study, we have also not found statistically significant arterial blood pressure differences in among the three groups.

In the magnesium group no patient suffered from shivering during this study, whereas shivering occurred in four (13.3%) patients belonging to control group and seven patients (33.3%) belonging to the clonidine group. The differences among groups were statistically significant (*P*<0.05). Perioperative magnesium supplementation prevents postoperative hypomagnesaemia and decreases the incidence of postoperative shivering.[3] Jeon *et al*.[23] observed that the intrathecal administration of clonidine 150 mcg fails to prevent postspinal shivering.

Sedation is a side effect frequently associated with the use of clonidine in postoperative analgesia, often in conjunction with opioids.[22] A bolus dose of epidural clonidine more than 100 mcg may cause significant sedation in women in labour.[24] But lower doses of clonidine will not cause significant sedation.[25]

Our study has the limitation of only one dose-response evaluation. We preferred to use a smaller dose of magnesium that would not cause any side-effects. In two cases reported by Goodman *et al*.,[26] larger doses (8.7 g, 9.6 g) of magnesium inadvertently administered into the epidural space did not cause any neurologic injury. Also another report described an inadvertent intrathecal injection of 1000 mg of magnesium producing a transient motor block followed by a complete resolution and no neurological deficit at long-term follow-up.[27] If larger doses are administered epidurally, does postoperative analgesic demand decrease or the analgesic effect enhance? Currently, the answer to this question is unknown. Only a study in rabbits reported toxicity with intrathecal magnesium used in larger doses,[28] and the hyperosmolar solutions of magnesium sulphate may have caused neurotoxicity. One of the main differences of this study from ours is the route of administration. And the second difference is the higher doses of magnesium they used in rabbits. We feel that further studies should address different dosages of magnesium with larger number of patients and different surgical settings to establish our findings.

## CONCLUSION

Co-administration of epidural magnesium with bupivacaine produces predictable rapid onset of surgical anaesthesia without any side-effects, and addition of clonidine to epidural bupivacaine produces prolonged duration of anaethesia with sedation. The results of the present investigation suggest that magnesium may be a useful alternative as an adjuvant to epidural bupivacaine.
